# The elevated NLR, PLR and PLT may predict the prognosis of patients with colorectal cancer: a systematic review and meta-analysis

**DOI:** 10.18632/oncotarget.18575

**Published:** 2017-06-19

**Authors:** Jie Zhang, Hong-Ying Zhang, Jia Li, Xin-Yu Shao, Chun-Xia Zhang

**Affiliations:** ^1^ Department of Oncology, The First Affiliated Hospital of Dalian Medical University, Dalian 116011, China; ^2^ Department of Pathology and Forensic Medicine, College of Basic Medical Sciences, Dalian Medical University, Dalian 116044, China; ^3^ Medical Oncology, The First Affiliated Hospital of Dalian Medical University, Dalian 116000, China

**Keywords:** colorectal cancer, neutrophils-to-lymphocytes ratio, platelet-to-lymphocytes ratio, platelet counts, prognosis

## Abstract

Recently, several studies have reported that inflammatory response and elevated platelet counts may be associated with the poor prognosis of colorectal cancer. This meta-analysis was designed to analyze and evaluate the prognostic role of elevated preoperative or pretreatment neutrophils-to-lymphocytes ratio, platelet-to-lymphocytes ratio or platelet counts in patients with colorectal cancer. We searched PubMed, EMBASE, Cochrane Library and Web of Science to April, 2016. A total of 23 studies (*N* = 11762 participants) were included for this meta-analysis. Elevated neutrophils-to-lymphocytes ratio have a close relationship with the poor Overall Survival of colorectal cancer with the pooled HR being 1.92 [95% CI 1.57–2.34; *P* < 0.00001]. This meta-analysis indicated that elevated neutrophils-to-lymphocytes ratio, platelet-to-lymphocytes ratio or platelet counts may be a cost-effective and noninvasive serum biomarker for poor prognosis for patients with colorectal cancer.

## INTRODUCTION

The colorectal cancer (CRC) is one of the increasingly common malignancies worldwide. According to the report from GLOBOCAN 2012, the incidence rate of CRC is at the third place becoming one of the most common cancers [1]. The incidence of the disease is 12.3% in men and 13.1% in women in Europe [2]. What is more, in North America, CRC is the third most common cause of cancer-related deaths [3]. At present, despite advanced instruments for screening and early detection of CRC, and surgical resection being the optimal option for the patients with colorectal cancer, about twenty percent of cases are found to be metastatic at the time of diagnosis and at least about half of patients die within 5 years after their diagnosis [4, 5]. Thus, appropriate prognostic markers were needed to predict patients’ postoperative prognosis and the survival of patients at high risk of recurrence, and to guide patients to choose additional treatment.

Hopefully, in recent years, several reviews have reported that elevated platelet counts and NLR may be associated with the poor prognosis of gastric cancer, lung cancer, renal cancer and gynecologic malignancies [6–9]. Besides, previous studies have showed that elevated NLR, PLR or PLT) also associated with poor prognosis for esophageal, renal and hepatocellular malignancies [10–14]. Likewise, the association between elevated NLR, PLR or PLT and the survival of colorectal cancer had also been published in some studies [15–48]. However, fallaciously, their results still remained inconsistent, with several studies drawing inverse conclusions [19, 22, 30, 49]. Therefore, we designed a meta-analysis based on relevant studies to analyze and evaluate the prognostic role of elevated preoperative or pretreatment NLR, PLR or PLT in patients with colorectal cancer.

## RESULTS

### Included studies and study characteristics

After 64 studies were excluded (54 studies for the cut-off value not meeting including criteria, 7 studies for lack of available data, 3 studies for just review), a total of 23 studies (*N* = 11762 participants) were included in this meta-analysis, of which 16 studies including 8691 participants were included for NLR [15, 36, 41, 43–45, 50–59], 6 studies including 1113 participants for PLR [15, 36, 50, 54, 60, 61] and 9 studies including 3685 participants for PLT [17, 21, 29, 31, 36, 42, 51, 56, 59, 62].

**Figure 1:**
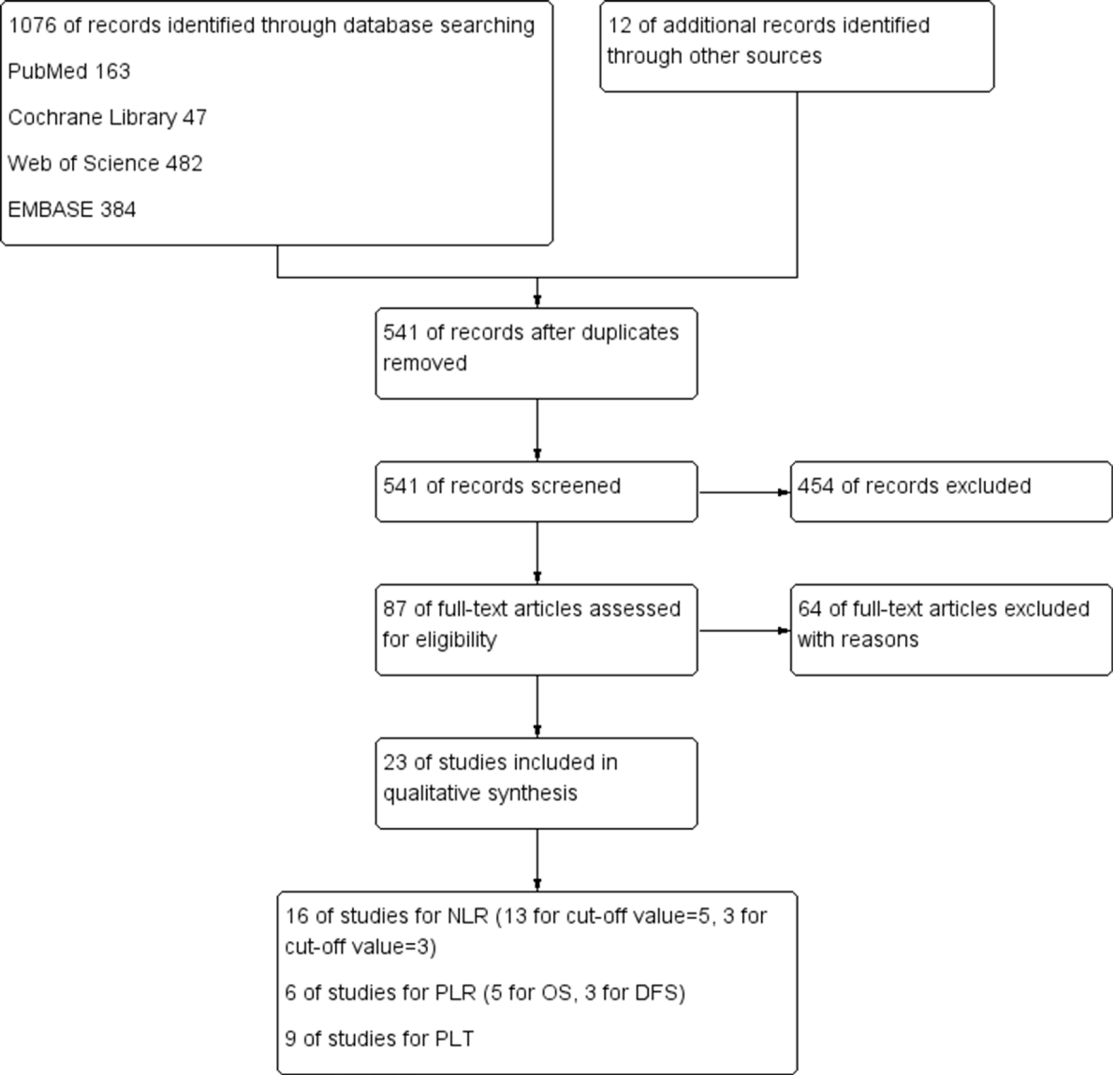
Flow diagram of the search strategy for pretreatment NLR, PLR and PLT as prognostic roles in patients with colorectal cancer

The detail search process and summary of studies were showed in study flow diagram (Figure 1). The other study characteristics were showed in Table 1.

**Table 1:** The characteristics of included studies for NLR, PLR and PLT

Year	Author	Country	Study design	Cut-off value	*N*	Clinical stage	*P* value	Pretreatment	FT (month)	NOS score	Outcome
The association between the prognosis and NLR in colorectal cancer patients:											
2015	Neal CP.	UK	Pro	5	302	NR	NR	Sur	29.7 (4—96)	6	OS
2014	Paik KY.	Korea	Pro	5	600	I — IV	0.402	Radio	27.4 (1—72)	8	OS
2014	Sun ZQ.	China	Pro	5	225	I — III	0.000	Sur	NR	6	OS, DFS
2013	Son HJ.	Korea	Retro	5	624	I — III	0.001	Sur	42.0 (1—66)	7	OS, DFS
2013	Urrejola G.	Chile	Pro	5	120	II	―	Sur	73.0 (20—114)	8	OS
2012	Carruthers R.	UK	Retro	5	115	NR	NR	CRT	37.1	6	OS, DFS
2012	Kwon HC.	Korea	Pro	5	200	I — IV	NR	Sur	NR	6	OS
2011	Chua W.	Australia	Pro	5	349	NR	NR	Chemo	NR	5	OS
2011	Hung HY.	Taiwan,	Pro	5	1040	I — II	―	Sur	74.5 (45.9—136.8)	7	OS, DFS
2009	Neal CP.	UK	Pro	5	174	NR	NR	Sur	NR	7	OS, DFS
2009	Kishi Y.	US	Pro	5	290	NR	NR	Sur, Chemo	28 (2—102)	8	OS
2008	Halazun KJ.	UK	Retro	5	440	NR	NR	Sur	24 (11—97)	6	OS
2007	Leitch EF.	UK	Pro	5	149	I — IV	0.001	Sur	48 (36—73)	8	OS
2012	Chiang SF.(C) *	Taiwan,C	Retro	3	1788	I — III	NR	Sur	96.2 (11.6—139.1)	7	OS
	Chiang SF.(R) *	Taiwan,C	Retro	3	1943	I — III	NR	Sur	96.2 (11.6—139.1)	7	OS
2013	He W.	China	Pro	3	243	NR	NR	Chemo	21.87	6	OS
2015	Toiyama Y.	Japan	Pro	3	89	I — III	NR	CRT	56 (2—147)	7	OS
The association between the prognosis and PLR in colorectal cancer patients:											
2015	Neal CP.	UK	Pro	150	302	NR	NR	Sur	29.7 (4—96)	6	OS
2014	Neofytou K.	UK	Retro	150	140	NR	NR	Chemo	33 (1—103)	7	OS
2014	Sun ZQ.	China	Pro	150	225	I — III	0.000	Sur	NR	6	OS, DFS
2015	Toiyama Y.	Japan	Pro	150	89	I — III	NR	CRT	56 (2—147)	7	OS, DFS
2015	Mori K.	Japan	Retro	150	157	I — III	0.176	Sur	20.5 (0.2—62.4)	7	DFS
2012	Kwon HC.	Korea	Pro	150	200	I — IV	NR	Sur	NR	6	OS
The association between the prognosis and PLT in colorectal cancer patients:											
2015	Josa V.	Hungary	Retro	400 × 109/L	336	I — IV	NR	Sur	46.0	6	OS
2015	Neal CP.	UK	Pro	400 × 109/L	302	NR	NR	Sur	29.7 (4—96)	6	OS
2014	Paik KY	Korea	Pro	400 × 109/L	600	I — IV	0.402	Radio	27.4 (1—72)	8	OS
2013	Wan S.	USA	Retro	400 × 109/L	1513	0 — IV	0.0001	Sur	46.7 (19.6—84.7)	7	OS
2010	Qiu Mz.	China	Retro	400 × 109/L	363	I — IV	0.001	NR	26 (3—50)	6	OS
2009	Neal CP.	UK	Pro	400 × 109/L	174	NR	NR	Sur	NR	7	OS, DFS
2007	Leitch EF.	UK	Pro	400 × 109/L	149	I — IV	0.001	Sur	48 (36—73)	8	OS
2005	Kandemir EG.	Turkey	Retro	400 × 109/L	198	NR	NR	Sur	47 (19—100)	6	OS
2012	Kaneko M.	Japan	Retro	400 × 109/L	50	NR	NR	Chemo	17.0 (0.77—61.6)	7	OS, DFS

* They were from the same study; the former represented colon cancer marked with C and the latter represented rectal cancer marked with R. N: total number of eligible patients. NOS: Newcastle-Ottawa Scale. OS: overall survival. DFS: disease free survival. FT: follow-up time (month) (median and range). Chemo: chemotherapy. Radio: radiotherapy. CRT: chemo-radiation. Sur: surgery. Retro: retrospective. Pro: prospective. NR: not report.

### The prognostic value of elevated NLR

Of the 16 studies included for NLR, 13 studies including 4628 participants provided the available data for evaluating the association between NLR (cut-off = 5) and OS, and 5 studies including 2178 participants for DFS. In addition, 3 studies [15, 43, 53] including 4063 participants were included for evaluating the association between NLR (cut-off value = 3) and OS.

**Figure 2:**
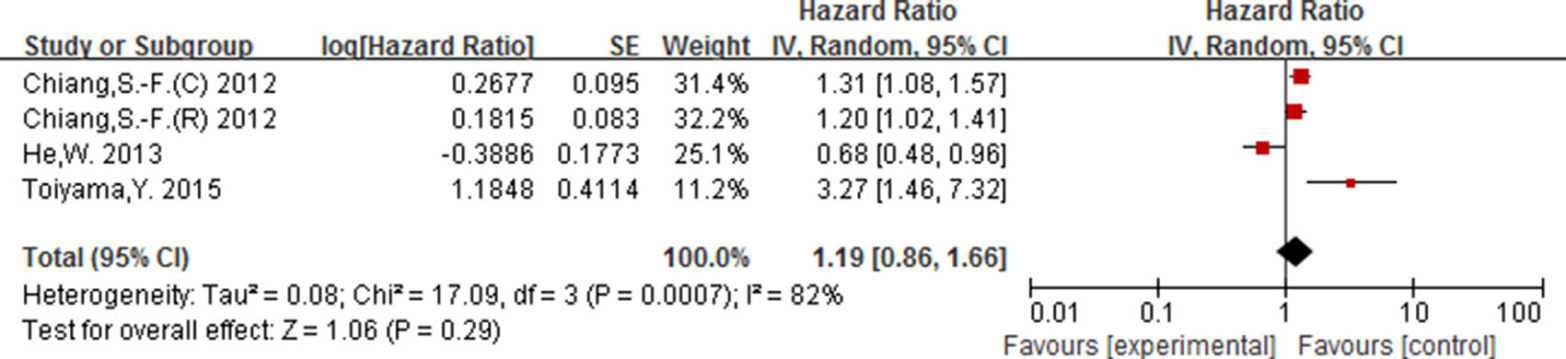
Forest plot of the prognostic effect of NLR (cut-off value = 3) on overall survival

**Figure 3:**
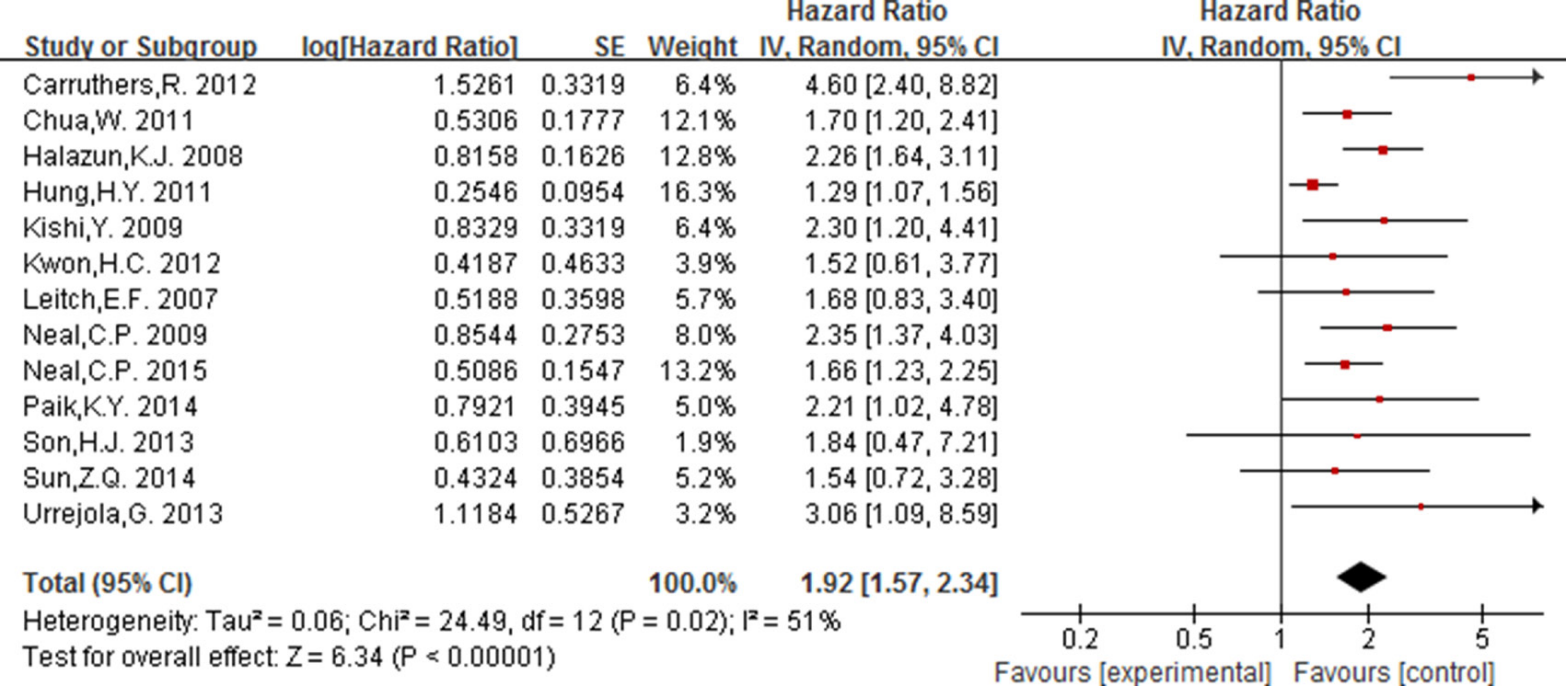
Forest plot of the prognostic effect of NLR (cut-off value = 5) on overall survival

**Figure 4:**
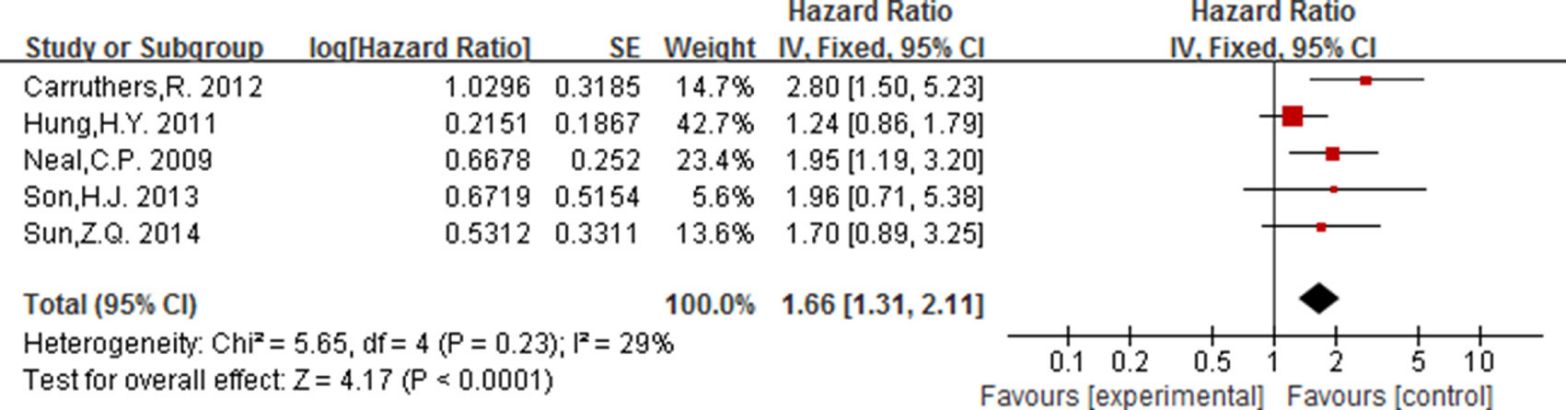
Forest plot of the prognostic effect of NLR (cut-off value = 5) on disease free survival

As displayed in Figure 2, for association between NLR (cut-off value = 3) and OS, meta-analysis showed no significant correlation, with the pooled HR being 1.19 [95% CI 0.86–1.66; *P* = 0.29]. For the cut-off value of NLR equal to 5, the pooled HR in patients with elevated NLR was significant higher than patients with normal NLR [HR = 1.92, 95% CI 1.57–2.34; *P* < 0.00001] (Figure 3). Significant results were also fund with the pooled analyses for the association between elevated NLR and DFS, with the pooled HR being 1.66 [95% CI 1.31–2.11; *P* < 0.0001] (Figure 4).

### The prognostic value of elevated PLR

Six studies reported the association between PLR (cut-off value = 150) and prognosis, of which five studies including 956 participants were for OS and three studies [15, 50, 60] including 471 participants were for DFS.

**Figure 5:**
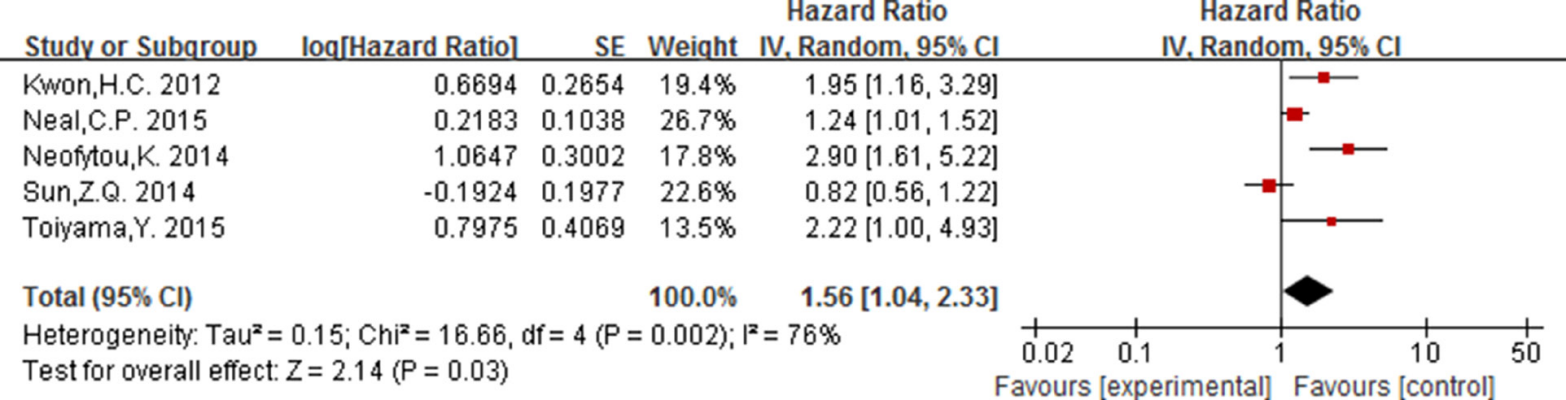
Forest plot of the prognostic effect of PLR (cut-off value = 150) on overall survival

**Figure 6:**
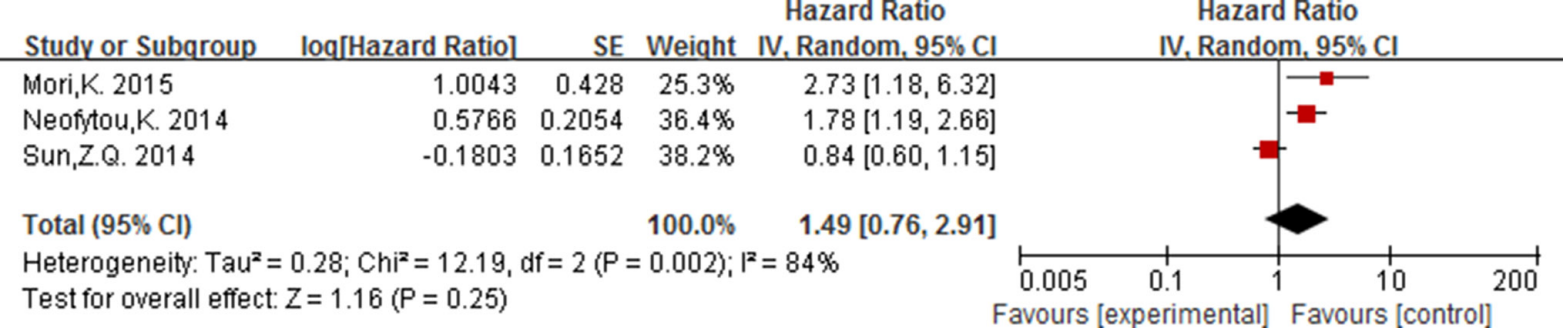
Forest plot of the prognostic effect of PLR (cut-off value = 150) on disease free survival

For PLR, as the significant heterogeneity between studies (*I^2^* ≥ 50%, *P ≤* 0.05), a random effect model was used to estimate the pooled HR. The combined HR indicated that elevated PLR had a statistically significant association with the OS of patients with colorectal cancer [HR = 1.56, 95% CI 1.04–2.33; *P* = 0.03] (Figure 5). The pooled HR with numerical significant heterogeneity indicated that elevated PLT had no significant association with DFS [HR = 1.49, 95% CI 0.76–2.91; *P* = 0.25] (Figure 6).

### The prognostic value of elevated PLT

**Figure 7:**
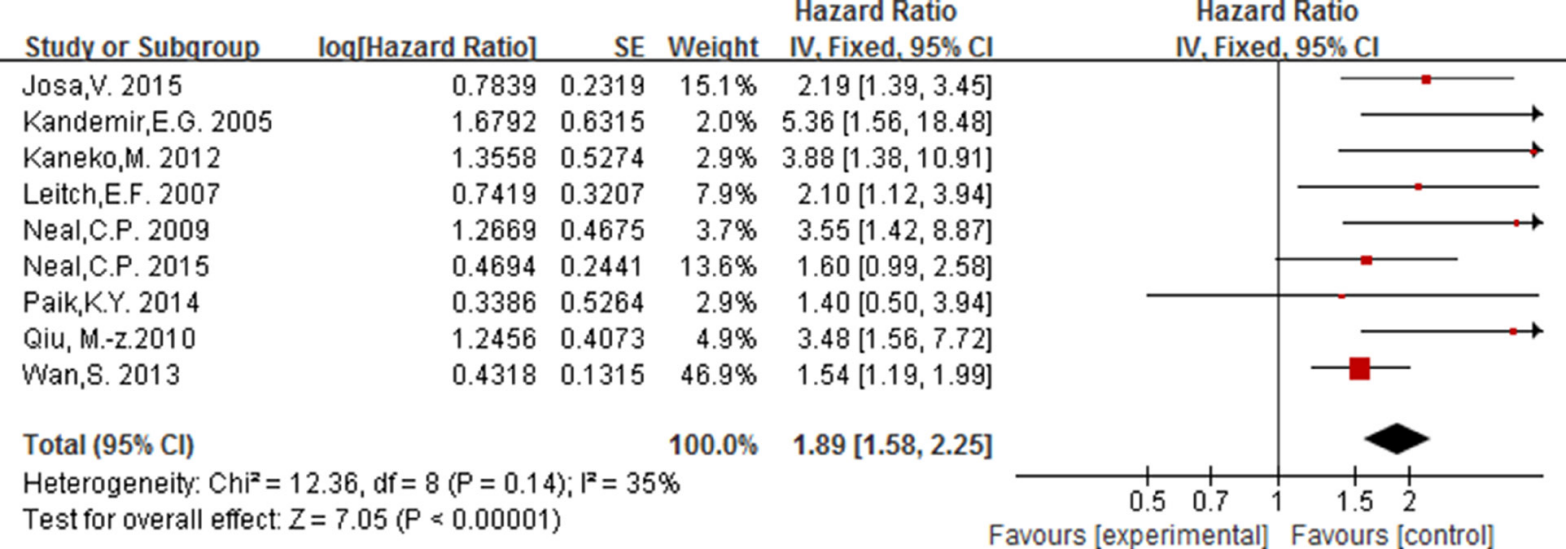
Forest plot of the prognostic effect of PLT (cut-off value = 400 × 10^9^/L) on overall

Nine studies including 3685 participants reported the association between elevated PLT and overall survival in patients with colorectal, with the cut-off value of platelet counts equal to 400 × 10^9^/L. As no significant heterogeneity between studies (*I^2^ ≤* 50% and *P* ≥ 0.05), a fixed effect model was used to estimate the association between elevated PLT and OS. The pooled HR with no significant heterogeneity indicated that elevated PLT had an obvious association with overall survival [HR = 1.89, 95% CI 1.58–2.25; *P* < 0.00001] (Figure 7).

### Publication bias

**Figure 8:**
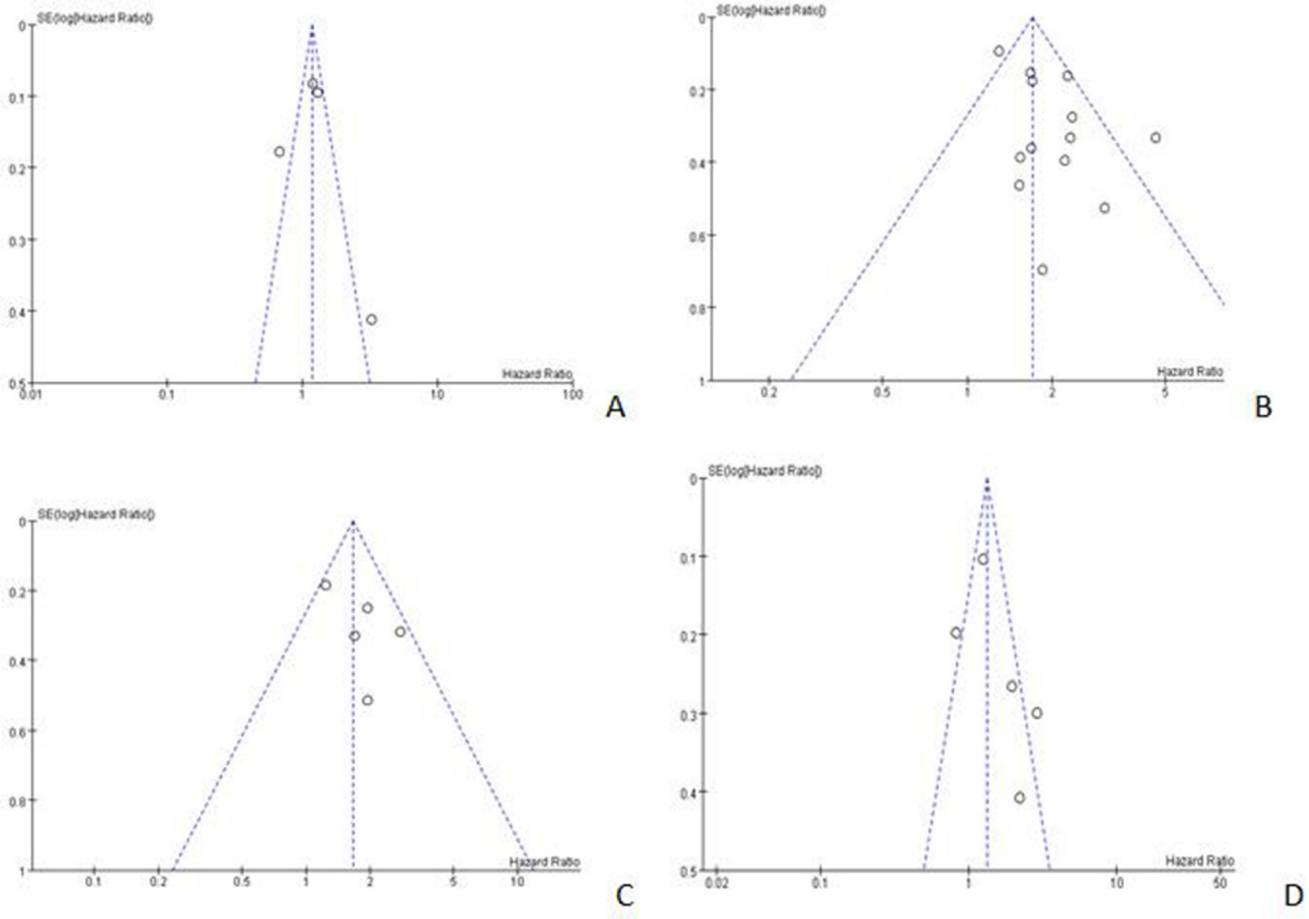
Begger's funnel plots for detecting publication bias (**A)** NLR (cut-off value = 3) for OS; (**B**) NLR (cut-off value = 5) for OS; (**C**) NLR (cut-off value = 5) for DFS; (**D**) PLR (cut-off value = 150) for OS.

Funnel plots were conducted for assessing the publication bias of included literatures and we could roughly assess the publication bias by seeing whether their shapes were of any obvious asymmetry. The funnel plots of NLR (cut-off value = 3) for OS, NLR (cut-off value = 5) for DFS and PLR did not reveal any obvious evidence of asymmetry (Figure 8A, 8C, 8D and Supplementary Figure 1). However, significant bias were found in both NLR (cut-off value = 5) and PLT for OS (Figure 8B and Supplementary Figure 2) and the analysis of causes were offered in discussion.

## DISCUSSION AND CONCLUSIONS

The relation between inflammation and malignancies was identified in the 19th century by Rudolf Virchow [63]. Growing evidences have support inflammation may play important roles in the development and progress of many inflammation, including CRC [14, 64, 65]. Many inflammatory cells and growth factors they produced could activate stroma and DNA-damage-promoting agents [66]. Meanwhile, tumors can increase inflammatory process and promote proliferation and metastases development of tumor cells by decreasing apoptosis and increasing angiogenesis and DNA damage in return [67].

Platelet plays an important role in the development and progression of malignancies as well. Great interest has been given in the prognostic role of thrombocytosis for patients with malignancies recently, as previously mentioned. However, the true mechanism or association between thrombocytosis and malignancies had not been clarified. There were several possible explanations for the association between elevated platelet counts and poor prognosis of malignancies. First, platelet might protect tumor cells from cytolysis, thereby promoting metastasis, by surface shielding them from immune system detection, and this seems to be the main mechanism of platelet protection [68]. Secondly, angiogenesis regulatory proteins are implicated in tumor growth and invasion. In colorectal cancer patients, the levels of PDGF, PF4 and VEGF are elevated in platelets, and the elevated levels of all three proteins correlated with the cancer state [69]. Platelets could stimulate angiogenic vessel growth and prevent hemorrhage from the angiogenic vessels, which was promoted by the adhesion function of platelet, as mediated by glycoprotein (GP) Ibα, and these processes could stimulate and potentiate tumor cells to form distant metastases [70]. Additionally, T-factor could reveal that platelets may assist tumor cells in invading to adjacent tissues, and thrombocytosis is just right strongly correlated with the progression of T-factor.

We perform this meta-analysis for purpose of demonstrating the indicative significance of NLR, PLR and elevated platelet counts for prognosis in patients with colorectal cancer. Our results statistically supported the conclusions that elevated NLR or PLT had a significant association with the poor prognosis in colorectal cancer, which was consist with the conclusions in gastric cancer, lung cancer and gynecologic malignancies [6, 7, 9]. In addition, we also assessed the association between elevated PLR and prognosis, but no significance was found, for the pooled HR being 1.56 [95% CI 1.04–2.33; *P* = 0.03] for OS and 1.49 [95% CI 0.76–2.91; *P* = 0.25] for DFS. By sensitivity analysis, we found that the study from Son, Z.Q 2014 had a significant impact for the results [50]. When the study was rejected from the analysis lists, the pooled HR became 1.86 [95% CI 1.20–2.89; *P* = 0.006] for OS and 1.93 [95% CI 1.34–2.77; *P* = 0.0004] for DFS. What is more, for the association between NLR (cut-off value = 3) and OS, meta-analysis also showed no significant correlation, with the pooled HR being 1.19 [95% CI 0.86–1.66; *P* = 0.29]. Likewise, when the study from He, Wen-zhuo. 2013 [53] was rejected by sensitivity analysis, the pooled HR became 1.36 [95% CI 1.05–1.75; *P* = 0.02]. Another cause of the results may be the small number patients or only a few studies for the analyses. The sensitivity analysis did not draw any different conclusions for other analyses in this meta-analysis.

Nevertheless, there were several limitations for this meta-analysis. The main limitation is the inconsistency of the cut-off values of NLR, PLR and platelet counts. For NLR, some studies used the cut-off values with 4, 2.9, 2.8, and 2.6 and so on. For example, Shen, L.J [39] suggested that 2.8 was the best cut-off value of NLR for distinguishing the prognosis of patients with CRC, differently from 4.0 in the study of Kaneko, M [42]. The cut-off values of PLR ranged from 150 to 300. But many studies used 150 as the best cut-off value. The definition of thrombocytosis or elevated platelet counts ranged from 260 × 10^9^/L to 400 × 10^9^/L. Based on the various discordance, we chose 5, 150 and 400 × 10^9^/L as the cut-off value of NLR, PLR and PLT respectively with decreasing the number of studies included. The second limitation is the variation of the clinical stages, for that the prognosis of patients with cancer is associated with clinical stages. The majority of studies included showed no significant difference in clinical stages with the *P* value of Chi-squared test more than 0.05. But there were many studies had significant differences in clinical stages for NLR [50, 52, 59], PLR [50] and platelet count [21, 29, 59] with the *P* value less than 0.05. Besides, the incidence or significance of the association between different clinical stages and prognosis for PLT was also different. The incidence of thrombocytosis was associated with clinical stages and increased to 12.2% and 20.6% in patients with stage III and IV disease respectively [19]. As 2014 Guo, Tian-hua reported, thrombocytosis has association with poor prognostic significance in patients with stage I to stage III colorectal cancer, but not in patients with stage IV disease. In addition, Sasaki K suggested that preoperative thrombocytosis was an independent indicator only in patients at stage II for cancer-specific survival [25]. Moreover, the time of measuring neutrophils, lymphocytes and platelet count before treatment or surgery might be an important limiting factor. Blood samples of patients were obtained within 5–7 days before surgery in the study of Sun, Z.Q. [50], but 2 weeks prior to operation in Kazuhito Sasaki [25]. Finally, the prognosis or the value of neutrophils, lymphocytes and platelet count were also influenced by other multiple factors, such as age, adjuvant therapy, and tumor size, histological type, venous involvement, which should also be taken into consideration. The last important limitation was the publication bias. As mentioned before, there were obvious bias in both NLR (cut-off value = 5) and PLT for OS and the analysis of causes were offered in discussion. The factors impacting publication bias may be various. In my opinion, apart from the factors like no publication of negative results, termination of publication, the language limitation may be the main influence, for that our searching language was only English.

Taking the limitations above into account, further researches need to clear the association between elevated NLR, PLR and platelet counts and prognosis in patients with different clinical stages.

Despite many difference and influencing factors, we could cautiously draw a conclusion that elevated NLR, PLR and platelet counts may have a close association with worse survival in colorectal cancer patients, with consideration of the evident statistical significance. Elevated NLR, PLR and platelet counts may be used as three prognostic indicators for early identification of CRC patients with worse prognosis, and then more attention and adjuvant therapy could be given to these patients.

## MATERIALS AND METHODS

### Including and excluding criteria

The including criteria of this meta-analysis were as follows: (1) All of randomized, controlled trials (RCTs), observational prospective or retrospective studies were included; (2) Included people with a diagnosis of colorectal cancer; (3) Included patients received operation or other adjuvant therapy; (4) Pretreatment platelet counts (cut-off value = 400 × 10^9^/L), NLR (cut-off value = 5) or PLR (cut-off value = 150) were reported or can be obtained; (5) The outcomes including OS or DFS were reported; (6) Sufficient data (HR and 95%CI on OS or DFS) were provided.

Excluding criteria were as follows: (1) Trials on animals; (2) Abstracts, letters, editorials, expert opinions, reviews, case reports; (3) Patients having other primary tumors; (4) Studies without sufficient data; (5) The cut-off value did not meet our including criteria.

### Search strategy

We searched PubMed, EMBASE, Cochrane Library and Web of Science to April, 2015. We also hand searched the citation lists of included studies and previously identified systematic reviews to identify further relevant trials. our searching terms and procedures were as follows: (1)“thrombocytosis”, “platelet count*”; (2) “neutrophil lymphocyte ratio”; “neutrophil to lymphocyte ratio”, NLR; (3) “platelet lymphocyte ratio”, “platelet to lymphocyte ratio”, PLR; (4) “colorectal cancer”, “rectal cancer”, “colonic cancer”; (5) “prognosis”, “survival”, “outcome”; (1 OR 2 OR 3) AND 4 AND 5. Other related terms, including references of some literatures we read, were also searched in English. Two assessors independently screened the titles and abstracts of each study. Once relevant studies became certain, the full texts were obtained for further evaluation.

### Quality assessment

Two reviewers assessed the quality of all the included studies using the 9-star Newcastle-Ottawa Scale (NOS) [71] independently, and the total scores of each study were displayed in the characteristics table. The scores were judged according to the three aspects of NOS of evaluation: selection, comparability, and outcome between the case group and control group. Studies with the scores ≥ 6 were assigned as high-quality studies.

### Data extraction

Data for the analysis were extracted independently by two reviewers, and disagreement was resolved by their discussion. In addition, the extracted contents including study demographics, published years, country, trial design, outcome and clinical stage were extracted using a standardized form.

Data collected were input into RevMan 5.2 software for analysis [72].

### Statistical analysis

In this meta-analysis, the impact of NLR, PLR or PLT on patients’ prognosis was measured by estimating the Hazard Ratio (HR) between elevated NLR, PLR or PLT groups and normal NLR, PLR or PLT groups. The associated 95% confidence intervals (CI) were also measured. The heterogeneity between studies was evaluated with *P* value and *I^2^*. *I^2^* ≥ 50% or *P ≤* 0.05 was deemed to represent significant heterogeneity [73, 74], and pooled HR was estimated using a Random-effect model. On the contrary, if statistical study heterogeneity was not observed (I^2^ ≤ 50% and *P* ≥ 0.05), a fixed effects model was used. Finally, publication bias was assessed by Begg's and Egger's test. If the shape of funnel plots revealed no obvious evidence of asymmetry, we considered that there was no obvious publication bias. All statistical analyses were performed using standard statistical procedures provided in RevMan 5.2 [73].

## SUPPLEMENTARY MATERIALS FIGURES


