# Supplementary material for: The elevated NLR, PLR and PLT may predict the prognosis of patients with colorectal cancer: a systematic review and meta-analysis
Source: Oncotarget. 2017 Jun 19;8(40):68837–46. doi: 10.18632/oncotarget.18575 (PMC5620300; doi:10.18632/oncotarget.18575)
Supplement: Supplementary file 1 [file oncotarget-08-68837-s001.pdf]

The elevated NLR, PLR and PLT may predict the prognosis of patients with colorectal cancer: a systematic review and meta-analysis

Supplementary Materials

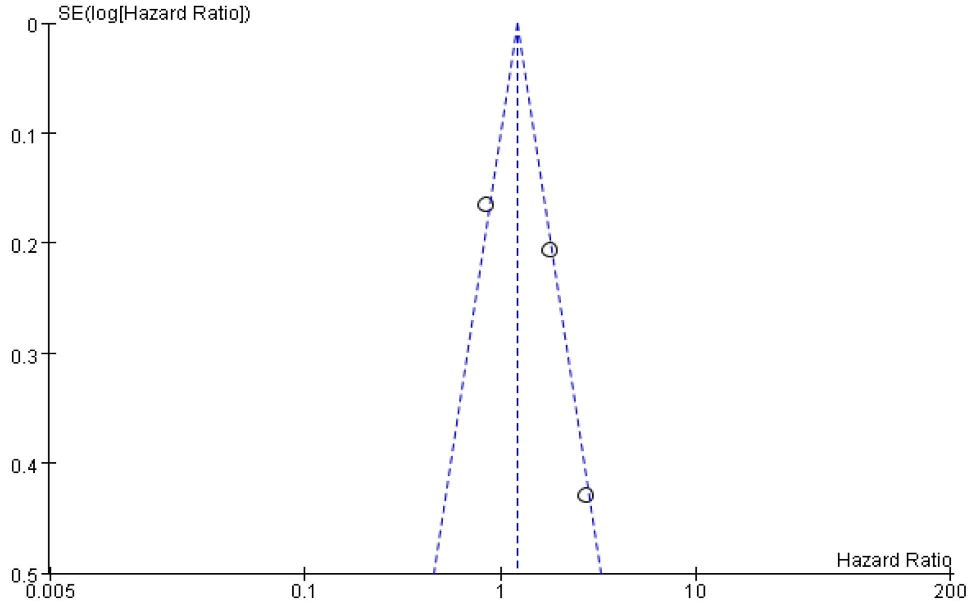

Supplementary Figure 1: Begger's funnel plots for detecting publication bias: PLR (cut-off value = 150) for DFS.

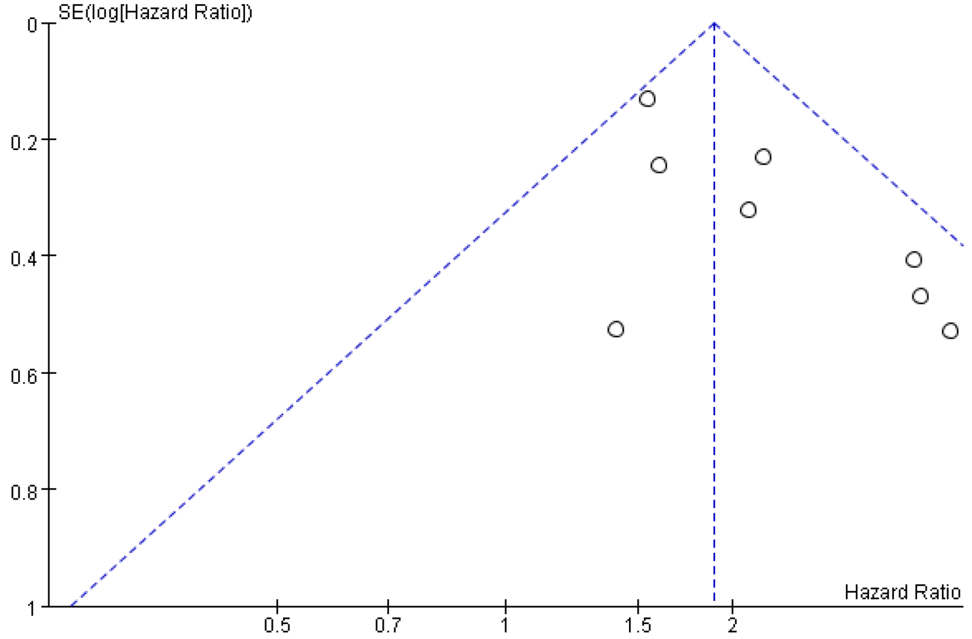

Supplementary Figure 2: Begger's funnel plots for detecting publication bias; PLT (cut-off value = 400 × 10^9/L) for OS.
